# Supplementary material for: Factors contributing to healthcare professional burnout during the COVID-19 pandemic: A rapid turnaround global survey
Source: PLoS One. 2020 Sep 3;15(9):e0238217. doi: 10.1371/journal.pone.0238217 (PMC7470306; doi:10.1371/journal.pone.0238217)
Supplement: S1 Questionnaire — (DOCX) [file pone.0238217.s001.docx]

**S1 Questionnaire. COVID-19 Questionnaire English Version.**

**Section 1: Demographics**

1. I am from: (Please write in Country)
2. I am a:
   1. Medical Student
   2. Nursing Student
   3. Medical Doctor (MD, DO)
   4. Physician Assistant, Nurse Practitioner, Certified Registered Nurse Anesthesiologist
   5. Licensed Nurse
   6. Pharmacist
   7. Administrative Staff
   8. Technologist or Technician
   9. Therapist
   10. I’m not a healthcare professional
3. Is your primary work in the Emergency Department (ER/UC), Intensive Care Unit (ICU), or Infectious Diseases (ID)?
   1. Yes
   2. No
4. Have you had any direct experience in working during any of the previous epidemics or pandemics? (SARS 2003, H1N1 2009, MERS 2012)
   1. Yes
   2. No

**Section 2: Personal exposure - since January 2020:**

1. Were you exposed to at least one person who has been diagnosed or had symptoms suggestive of COVID-19 infection?
   1. Yes
   2. No
2. How many people were you exposed to?
   1. 1-10
   2. 11-100
   3. More than 100
3. Have you experienced flu-like symptoms or symptoms suggestive of COVID-19 infection?
   1. Yes
   2. No
4. Did you stop working after experiencing these symptoms?
   1. Yes
   2. No
5. What did you do after you developed symptoms suggestive of COVID-19? (Select all that apply)
   1. I voluntarily self-quarantined
   2. I voluntarily kept working
   3. My institution told me to stay at home
   4. My institution told me to keep working
   5. I physically distanced myself from my family / loved ones
   6. I went to see a doctor
   7. I called a doctor
   8. I went to the Emergency Room/Hospital
   9. I started taking medications
6. Were you tested for COVID-19?
   1. Yes
   2. No
7. How easy was it to get tested for COVID-19?
   1. Very difficult
   2. Difficult
   3. Not difficult or easy
   4. Easy
   5. Very easy
8. How many times were you tested for COVID-19?
   1. 0
   2. 1 - 2
   3. 3 - 4
   4. 5+
9. Did you test positive for COVID-19??
   1. Yes
   2. No

**Section 3: Perception - since January 2020:**

1. What is your current perception of the COVID-19 disease?
   1. Benign disease
   2. Mild disease
   3. Moderate disease
   4. Severe disease
2. Has your perception of the COVID-19 virus changed?
   1. Yes
   2. No
3. Do you think that, during the initial days, you underestimated the public health effects of the pandemic?
   1. Yes
   2. No
4. Have you had any specific training related to the COVID-19 pandemic?
   1. Yes
   2. No
5. Do you feel this training was sufficient?
   1. Yes
   2. No
6. Did you receive appropriate guidelines on updated procedures related to personal safety to follow at work?
   1. Yes
   2. No
7. Did your institution provide you adequate PPE? (personal protective equipment)
   1. Yes
   2. No
8. What changes did you apply in your personal protection after COVID-19 affected hit your country? (Select all that apply)
   1. I started wearing masks routinely
   2. I started using hand sanitizer/washing my hands more frequently
   3. I started wearing more scrubs/disposable clothes
   4. I started social distancing
   5. Other. Please specify __________________________
9. Did you decide NOT to wear a mask or other PPE when it was available? (Select all that apply)
   1. No, never
   2. Yes, for fear of scaring patients
   3. Yes, for fear of scaring my colleagues
   4. Yes, because none of my colleagues were wearing it
   5. Yes, because my colleagues told me not to wear it
   6. Yes, because my institution didn’t recommend it
   7. Yes, because my institution told me not to wear it
   8. Yes, because it is uncomfortable/inconvenient
   9. Yes, because I didn’t believe I needed to wear it
   10. Yes, for other reasons. Please specify: ___________________________________
10. If you are fit and well during the COVID-19 pandemic, how likely are you to continue working?
    1. Extremely unlikely
    2. Unlikely
    3. Neutral
    4. Likely
    5. Extremely likely
11. Some people think that healthcare workers have unconditional obligations to work, even when the risks to themselves are great. To what extent do you agree with this statement?
    1. Strongly disagree
    2. Disagree
    3. Somewhat disagree
    4. Neither agree or disagree
    5. Somewhat agree
    6. Agree
    7. Strongly agree
12. What worries do you have about working during a pandemic? (Select all that apply)
    1. Becoming sick
    2. Making my loved ones sick
    3. Not having the appropriate skills to care for COVID-19 patients
    4. Not having adequate supplies to care for COVID-19 patients
    5. Other: (please write in)
13. Has your institution made psychological or mental health support available to you?
    1. Yes
    2. No
14. Are patients refusing medical services or missing appointments for fear of being infected?
    1. Yes
    2. No
15. Do you think that telemedicine-based clinical encounters are an effective way to replace in-person clinical encounters?
    1. Yes
    2. No
16. Do you think postponing non-urgent health issues will fasten the resolution of the COVID-19 pandemic?
    1. Yes
    2. No
17. Do you feel you are being pushed beyond your training?
    1. Yes
    2. No
18. Have you been redirected to activities related to COVID-19?
    1. Yes
    2. No
19. How long have you been engaged in COVID-19 related activities?
    1. 1-30 days,
    2. 31-60 days,
    3. 61-90 days
    4. 91+ days
20. Did you experience a moment whereby you had to make a prioritizing decision about vital issues (ICU admission, intubation, etc.) due to shortage of medical supplies?
    1. Yes
    2. No
21. Which of the following factors would or did influence your decision to prioritize treatment of certain COVID-19 patient groups over others, due to the shortage of medical supplies?
    1. age
    2. chronic medical conditions
    3. presenting symptoms
    4. clinical reasoning
    5. other (please specify)

**Section 4: Workload - compared to your average workweek, in the last 7 days:**

1. How many additional/fewer hours did you work?
   1. 30+ hours per week **less** than usual
   2. 16-30 hours per week **less** than usual
   3. 1-15 hours per week **less** than usual
   4. **Same** hours per week as usual
   5. 1-15 hours per week **more** than usual
   6. 16-30 hours per week **more** than usual
   7. 30+ hours per week **more** than usual
2. Was this change voluntary or mandatory?
   1. Voluntary
   2. Mandatory
3. How have your currently daily duties impacted your quality of life, compared to your duties before the pandemic?
   1. Much better quality of life
   2. Somewhat better quality of life
   3. Same quality of life
   4. Somewhat worse quality of life
   5. Much worse quality of life
4. How has work affected your ability to perform household activities? (Household activities include but are not limited to: childcare, grocery shopping, laundry, cleaning, cooking, etc.)
   1. Not at all
   2. To a small extent
   3. To some extent
   4. To a moderate extent
   5. To a great extent
   6. To a very great extent
5. If you have additional free time, how are you spending it? (Select all that apply)
   1. Spending time with my family/friends
   2. Self-education
   3. Cooking
   4. Working out
   5. Social media
   6. Watching movies/TV series
   7. I have the same or less free time
6. To what extent do you agree with the following statement: I am burned out from my work.
   1. Strongly disagree
   2. Disagree
   3. Somewhat disagree
   4. Neither agree or disagree
   5. Somewhat agree
   6. Agree
   7. Strongly agree
