# Supplementary material for: Factors contributing to healthcare professional burnout during the COVID-19 pandemic: A rapid turnaround global survey
Source: PLoS One. 2020 Sep 3;15(9):e0238217. doi: 10.1371/journal.pone.0238217 (PMC7470306; doi:10.1371/journal.pone.0238217)

**S2 Figure. Predictors of burnout in HICs and LMICs.** Predictors of burnout in HICs (above) and LMICs (below). (PPE) Personal protective equipment.


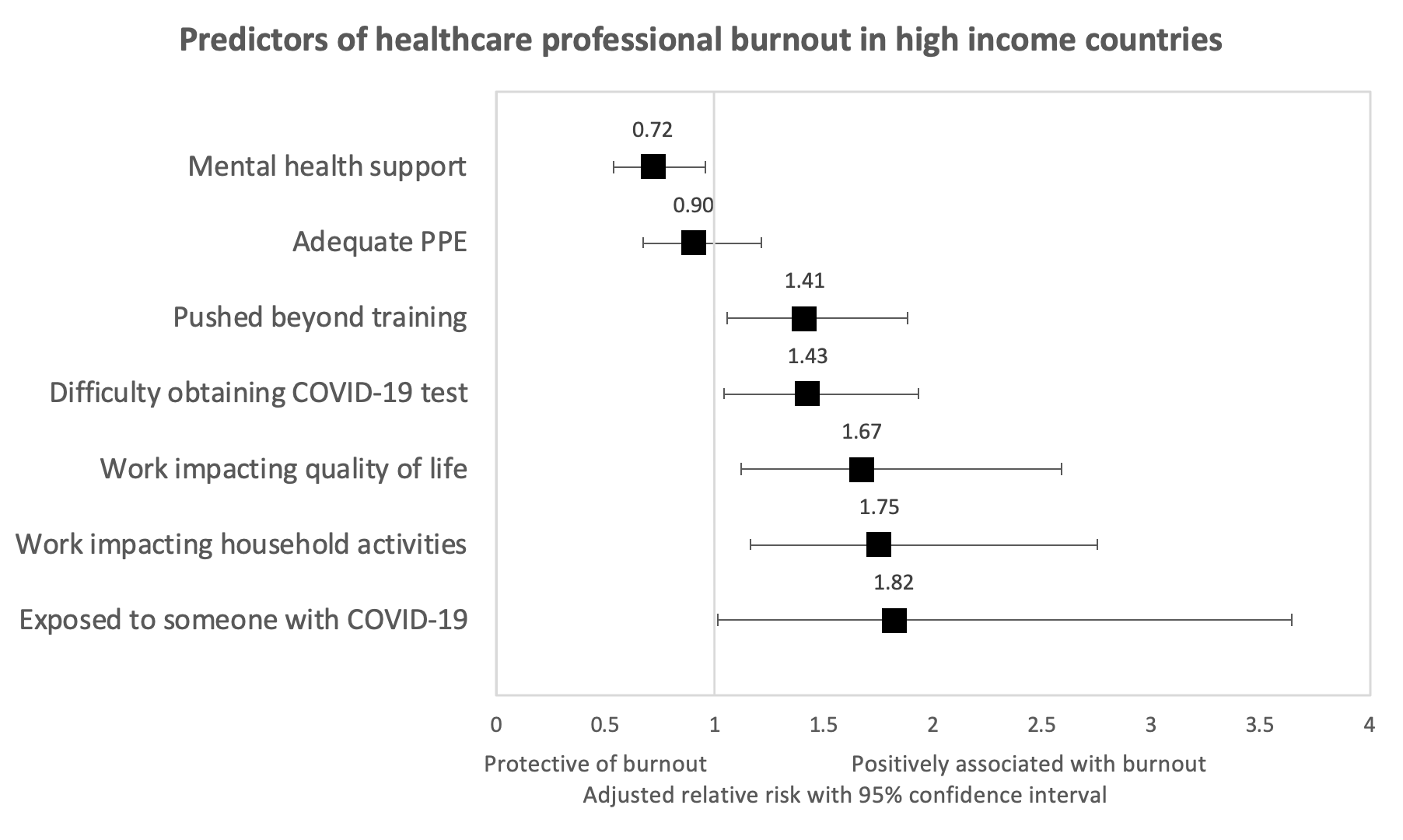


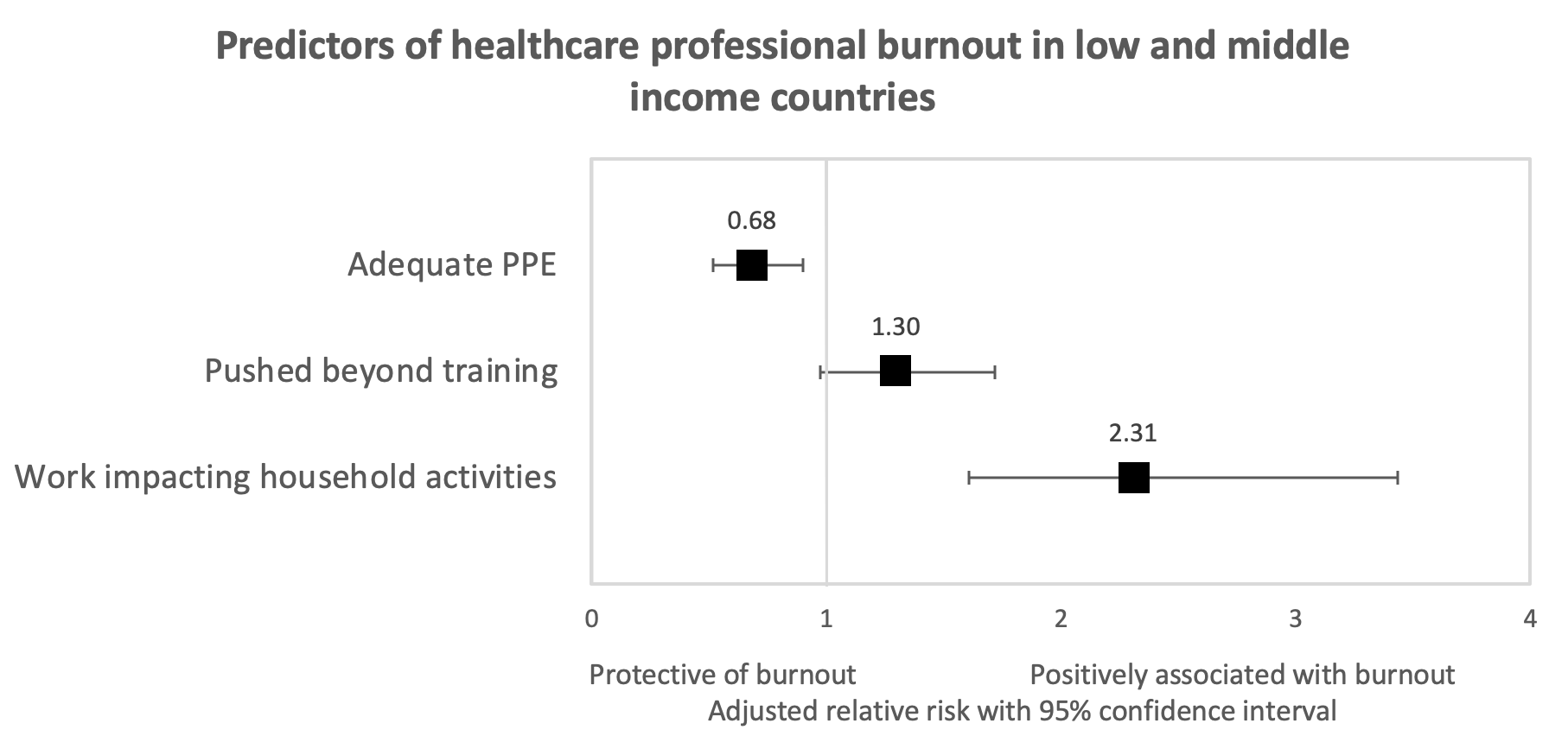

Supplement: S1 Fig — Predictors of burnout in HICs (above) and LMICs (below). (PPE) Personal protective equipment. (DOCX) [file pone.0238217.s002.docx]
