# Supplementary material for: Factors contributing to healthcare professional burnout during the COVID-19 pandemic: A rapid turnaround global survey
Source: PLoS One. 2020 Sep 3;15(9):e0238217. doi: 10.1371/journal.pone.0238217 (PMC7470306; doi:10.1371/journal.pone.0238217)
Supplement: S1 Table — Conversion of ordinal variables into binary; (QoL) Quality of life; (PA) Physician assistant; (NP) Nurse practitioner; (CRNA) Certified registered nurse anesthetist; (RN) Registered nurse. (DOCX) [file pone.0238217.s003.docx]

**S3 Table. Conversion of ordinal variables into binary.** Conversion of ordinal variables into binary; (QoL) Quality of life; (PA) Physician assistant; (NP) Nurse practitioner; (CRNA) Certified registered nurse anesthetist; (RN) Registered nurse.

| Original ordinal variable | Combined levels to binary |
| --- | --- |
| Burnout | Burnout binary:  Strongly disagree -> Burnout No  Disagree -> Burnout No  Somewhat disagree -> Burnout No  Neither agree nor disagree -> Burnout No  Somewhat agree -> Burnout Yes  Agree -> Burnout Yes  Strongly agree -> Burnout Yes |
| Work effect on QoL | Much better quality of life -> Lower QoL NO  Somewhat better quality of life -> Lower QoL NO  Same quality of life -> Lower QoL NO  Somewhat worse quality of life -> Lower QoL YES  Much worse quality of life -> Lower QoL YES |
| Change in workload | 30+ hours per week less than usual -> Larger workload No  16-30 hours per week less than usual -> Larger workload No  1-15 hours per week less than usual -> Larger workload No  Same hours per week as usual -> Larger workload No  1-15 hours per week more than usual -> Larger workload Yes  16-30 hours per week more than usual -> Larger workload Yes  30+ hours per week more than usual -> Larger workload Yes |
| Work effect on household activities | Not at all -> NO  To a small extent -> NO  To some extent -> YES  To a moderate extent -> YES  To a great extent -> YES  To a very great extent -> YES |
| Number of COVID-19 exposures | 0 Exposed NO  1-10 Exposed YES  11-100 Exposed YES  101 or more Exposed YES |
| Difficulty getting tested for COVID-19 | Very difficult Difficult YES  Difficult Difficult YES  Neutral Difficult NO  Easy Difficult NO  Very easy Difficult NO |
| Occupation | “Medical Doctors”, “Resident or Fellow” -> ”Medical Doctors”  “PA, NP, CRNA, RN” -> “Nurses”  “Others” -> “Others” |
| Provenience | US, Italy, Brazil, Sweden, Others |
