# Supplementary material for: Factors contributing to healthcare professional burnout during the COVID-19 pandemic: A rapid turnaround global survey
Source: PLoS One. 2020 Sep 3;15(9):e0238217. doi: 10.1371/journal.pone.0238217 (PMC7470306; doi:10.1371/journal.pone.0238217)
Supplement: S2 Table — Country of provenience of study participants and classification according to the World Bank. (DOCX) [file pone.0238217.s004.docx]

**S4 Table. Country of provenience of study participants and gross domestic product (GDP) information.** Country of provenience of study participants and classification according to the World Bank.

| Country | Responses | Percentage | World Bank Classification |
| --- | --- | --- | --- |
| Afghanistan | 1 | 0.04 % | Low income |
| Albania | 1 | 0.04 % | Upper Middle income |
| Algeria | 2 | 0.09 % | Upper Middle income |
| Argentina | 55 | 2.43 % | Upper Middle income |
| Australia | 11 | 0.49 % | High income |
| Bangladesh | 5 | 0.22 % | Low-Middle income |
| Bhutan | 1 | 0.04 % | Low-Middle income |
| Bolivia | 1 | 0.04 % | Low-Middle income |
| Brazil | 188 | 8.29 % | Upper Middle income |
| Burundi | 1 | 0.04 % | Low income |
| Canada | 14 | 0.62 % | High income |
| Chile | 2 | 0.09 % | High income |
| China | 57 | 2.51 % | Upper Middle income |
| Colombia | 7 | 0.31 % | Upper Middle income |
| Denmark | 2 | 0.09 % | High income |
| Dominica | 1 | 0.04 % | Upper Middle income |
| Ecuador | 3 | 0.13 % | Upper Middle income |
| Egypt | 4 | 0.18 % | Low-Middle income |
| El Salvador | 3 | 0.13 % | Low-Middle income |
| Ethiopia | 1 | 0.04 % | Low income |
| Finland | 2 | 0.09 % | High income |
| France | 16 | 0.71 % | High income |
| Georgia | 1 | 0.04 % | Upper Middle income |
| Germany | 12 | 0.53 % | High income |
| Greece | 4 | 0.18 % | High income |
| Guatemala | 1 | 0.04 % | Upper Middle income |
| Hungary | 1 | 0.04 % | High income |
| Iceland | 1 | 0.04 % | High income |
| India | 16 | 0.71 % | Low-Middle income |
| Indonesia | 2 | 0.09 % | Low-Middle income |
| Iran | 5 | 0.22 % | Upper Middle income |
| Ireland | 1 | 0.04 % | High income |
| Israel | 3 | 0.13 % | High income |
| Italy | 610 | 26.91 % | High income |
| Japan | 1 | 0.04 % | High income |
| Latvia | 1 | 0.04 % | High income |
| Lebanon | 1 | 0.04 % | Upper Middle income |
| Lithuania | 1 | 0.04 % | High income |
| Mexico | 10 | 0.44 % | Upper Middle income |
| New Zealand | 1 | 0.04 % | High income |
| Norway | 1 | 0.04 % | High income |
| Peru | 2 | 0.09 % | Upper Middle income |
| Philippines | 4 | 0.18 % | Low-Middle income |
| Poland | 15 | 0.66 % | High income |
| Puerto Rico | 1 | 0.04 % | High income |
| Romania | 1 | 0.04 % | Upper Middle income |
| Russia | 1 | 0.04 % | Upper Middle income |
| Slovakia | 2 | 0.09 % | High income |
| South Korea | 36 | 1.59 % | High income |
| Spain | 5 | 0.22 % | High income |
| Sweden | 149 | 6.57 % | High income |
| Switzerland | 1 | 0.04 % | High income |
| Syria | 2 | 0.09 % | Low income |
| Thailand | 29 | 1.28 % | Upper Middle income |
| Turkey | 100 | 4.41 % | Upper Middle income |
| Ukraine | 5 | 0.22 % | Low-Middle income |
| United Kingdom | 22 | 0.97 % | High income |
| United Nations | 3 | 0.13 % | High income |
| United States | 836 | 36.88 % | High income |
| Venezuela | 1 | 0.04 % | Upper Middle income |
| Vietnam | 1 | 0.04 % | Low-Middle income |
