# Supplementary material for: Factors contributing to healthcare professional burnout during the COVID-19 pandemic: A rapid turnaround global survey
Source: PLoS One. 2020 Sep 3;15(9):e0238217. doi: 10.1371/journal.pone.0238217 (PMC7470306; doi:10.1371/journal.pone.0238217)
Supplement: S3 Table — Healthcare professionals responses to perception, exposure, and workload during the COVID-19 pandemic. (HCP) Healthcare professional; (PPE) Personal protective equipment; (QoL) Quality of life; (ICU) Intensive care unit; (ER) Emergency room; (ID) Infectious diseases; (CRNA) Certified registered nurse anesthetist. (DOCX) [file pone.0238217.s005.docx]

**S5 Table. Additional survey responses.** Healthcare professionals’ responses to perception, exposure, and workload during the COVID-19 pandemic. (HCP) Healthcare professional; (PPE) Personal protective equipment; (QoL) Quality of life; (ICU) Intensive care unit; (ER) Emergency room; (ID) Infectious diseases; (CRNA) Certified registered nurse anesthetist.

| Occupation specific |  |
| --- | --- |
| Medical student | 47 (2.3%) |
| Nursing Student | 8 (0.4%) |
| Resident or Fellow | 160 (8.0%) |
| Medical Doctor (MD, DO) | 719 (35.9%) |
| Physician Assistant, Nurse Practitioner, CRNA | 36 (1.8%) |
| Registered Nurse | 855 (42.7%) |
| Pharmacist | 22 (1.1%) |
| Administrative staff | 42 (2.1%) |
| Technologist or technician | 59 (2.9%) |
| Not a healthcare professional | 54 (2.7%) |
| Primary work in the ICU/ER/ID prior to pandemic |  |
| No | 1382 (68.8%) |
| Yes | 628 (31.2%) |
| Previous experience working during pandemic |  |
| No | 1511 (75.0%) |
| Yes | 504 (25.0%) |
| Number of COVID-19 exposures |  |
| 1-10 | 787 (62.8%) |
| 11-100 | 401 (32.0%) |
| More than 100 | 66 (5.3%) |
| Difficulty obtaining a test |  |
| Very difficult | 20 (7.4%) |
| Difficult | 42 (15.5%) |
| Neutral | 75 (27.7%) |
| Easy | 87 (32.1%) |
| Very easy | 47 (17.3%) |
| Number of times tested for COVID-19 |  |
| 0 | 1611 (85.4%) |
| 1-2 | 251 (13.3%) |
| 3-4 | 19 (1.0%) |
| 5+ | 6 (0.3%) |
| Has perception changed |  |
| No | 681 (39.2%) |
| Yes | 1056 (60.8%) |
| initial underestimation of severity |  |
| No | 597 (34.4%) |
| Yes | 1137 (65.6%) |
| Was COVID-19 training sufficient |  |
| No | 425 (52.3%) |
| Yes | 388 (47.7%) |
| Did you receive guidelines on COVID-19 personnel safety |  |
| No | 513 (29.6%) |
| Yes | 1219 (70.4%) |
| Will keep working during pandemic if fit enough |  |
| Extremely unlikely | 48 (2.8%) |
| Unlikely | 51 (3.0%) |
| Neutral | 131 (7.6%) |
| Likely | 301 (17.5%) |
| Extremely likely | 1193 (69.2%) |
| HCP has unconditional obligation to work |  |
| Strongly disagree | 323 (18.7%) |
| Disagree | 292 (16.9%) |
| Somewhat disagree | 211 (12.2%) |
| Neither agree nor disagree | 122 (7.1%) |
| Somewhat agree | 358 (20.7%) |
| Agree | 280 (16.2%) |
| Strongly agree | 141 (8.2%) |
| Patients not getting care out of fear of being infected |  |
| No | 332 (19.4%) |
| Yes | 1379 (80.6%) |
| Think telemedicine is effective |  |
| No | 662 (38.4%) |
| Yes | 1062 (61.6%) |
| Postponing non-urgent care will fasten resolution of COVID-19 |  |
| No | 386 (22.3%) |
| Yes | 1343 (77.7%) |
| Redirected to COVID-19 related care |  |
| No | 950 (55.2%) |
| Yes | 772 (44.8%) |
| Length of COVID-19 care (days) |  |
| 1-30 days | 507 (65.8%) |
| 31-60 days | 234 (30.4%) |
| 61-90 days | 23 (3.0%) |
| 91+ days | 6 (0.8%) |
| Rationale for life-prioritizing decision |  |
| Age | 276 (18.5%) |
| Chronic medical conditions | 316 (21.2%) |
| Presenting symptoms | 238 (16.0%) |
| Clinical reasoning | 536 (36.0%) |
| Other | 122 (8.2%) |
| Mandatory or voluntary COVID-19 work |  |
| Voluntary | 801 (52.1%) |
| Mandatory | 737 (47.9%) |
