# Supplementary material for: Factors contributing to healthcare professional burnout during the COVID-19 pandemic: A rapid turnaround global survey
Source: PLoS One. 2020 Sep 3;15(9):e0238217. doi: 10.1371/journal.pone.0238217 (PMC7470306; doi:10.1371/journal.pone.0238217)
Supplement: Attachment — Submitted filename: Review 1.docx [file pone.0238217.s007.docx]

# General

- Limited questionnaire, but not much that can be done about this
- Needs multivariate regression, including demographic variables

# Abstract

- Geographical spread is impressive
- Method needs to state definition of burnout

# Research in Context

- The ‘Evidence before this study’ merely states a search strategy without stating what the established knowledge was before the study was conducted

# Introduction

- This is a minor point. The authors state in the first sentence ‘novel coronavirus (COVID-19)’. The name of the novel coronavirus is SARS-CoV-2, whereas the name of the disease is COVID-19. This should be corrected.

# Methods

- The scope of the study in terms of geography and translation into 18 languages is impressive
- It is a shame that validated self-report rating scales were not used, rather than 40 questions based on expert opinion
- Were demographic data collected from respondents? This is not mentioned in the Methods.
- I am not familiar with the burnout literature, but having the main outcome as a single question on self-report burnout seems like an unstable measure that would show high intra-individual variability. It would have been more helpful to use a validated tool, e.g. as listed here: <https://nam.edu/valid-reliable-survey-instruments-measure-burnout-well-work-related-dimensions/>
- Only participants who responded completely were included in the regression were included. This is reasonable, but it is important to assess whether completers differed from non-completers in any important ways.
- Before participants were asked whether they were ‘burned out’ by their work, were they given any definition of being ‘burned out’?

# Results

- 2707 valid responses were received. The manuscript should state how many invalid responses were received.
- Bivariate associations were reported between burnout and various other factors. It would be important to see a multivariate analysis to see which factors were independently associated with burnout.
- In particular, I cannot see any attempt made to examine demographic associations with burnout. These need to be included in a regression model, as they might be significant confounders.

# Discussion

- The 3^rd^ sentence mentions factors that typically increase the likelihood of HCP burnout. This needs a citation.
- The 2^nd^ paragraph rather overreaches itself by stating that burnout could be ‘prevented or minimised’. Given that burnout is always prevalent at a certain rate, it is highly improbable that it could be prevented. Minimisation of burnout may be possible, but this is not demonstrated by this paper, which does not examine interventions. This must be stated more cautiously.
- Paragraph 3 states that ‘HCPs who worked extensively during the SARS pandemic in Beijing later demonstrated posttraumatic stress symptoms’. Surely this is only some HCPs.
