# Supplementary material for: Factors contributing to healthcare professional burnout during the COVID-19 pandemic: A rapid turnaround global survey
Source: PLoS One. 2020 Sep 3;15(9):e0238217. doi: 10.1371/journal.pone.0238217 (PMC7470306; doi:10.1371/journal.pone.0238217)
Supplement: Attachment — Submitted filename: Response to Reviewers letter.docx [file pone.0238217.s008.docx]

Michio Murakami

Academic Editor

PLOS ONE

RE: PONE-D-20-13536, “Factors Contributing to Healthcare Professional Burnout During the COVID-19 Pandemic: A Rapid Turnaround Global Survey”

July 23^rd^, 2020

Dear Dr. Murakami,

Thank you for your thoughtful review of our manuscript. We have carefully reviewed the Reviewer’s comments and used them to prepare a revised manuscript for your consideration.

Below, we summarize the reviewer’s concerns and our responses.

**Journal Requirements**

1. We modified the manuscript to follow PLOS ONE’s style requirements, including those for file naming. Thank you for providing the PLOS ONE style templates.
2. The Acknowledgements section of the manuscript has been modified to not include the funding information that was previously mentioned. The authors received no specific funding for this work, thus the Funding Statement has not been modified.
3. The manuscript submission data has been amended to include author Heng Wang instead of Heng Want.
4. Captions for the Supporting Information files have been included at the end of the manuscript and in-text citations have been updated according to PLOS ONE guidelines.

**Additional Editor Comments**

1. We were advised by the Editor and the Reviewers to explain in more detail the recruitment method and the participant population. In particular, we edited the “Sample Population” paragraph in the Methods section, replacing it with “Sample Population and Recruitment Strategy” and including the requested information.
2. We were asked by the Editor to describe the validity and reliability of the outcome. We acknowledge in the Limitations section that our survey has not been validated in its entirety. The emotional exhaustion burnout outcome, which was measured with a single item, has been successfully validated in a cited article (doi:10.1007/s11606-009-1129-z). We recognize how the multifaceted nature of burnout cannot be simplified with a single question but we do believe that in the peak phase of the pandemic a single item question would have been more agile for HCPs to reply when compared to the 22-item MBI, thus increasing response rates.

We also recognize how emotional exhaustion represents the only core burnout domain noted to be generalizable across cultures, while depersonalization and personal accomplishment are Western concepts that cannot be applied to other societies (doi 10.1007/978-3-319-52887-8_5 pages 118-121).

3. The Editor expressed concern that our comparison of reported burnout levels (51.4%) with another study (40%) could be inappropriate. We agree with the comment: the comparison of reported burnout as measured with the 22-item MBI against our single item would not be appropriate. We do not make such comparisons. Nonetheless, we still believe that it is valuable to describe the previously reported rates and we suggest future studies to unpack potential differences. It seems justifiable to comment on possible factors that may drive the differences.
4. The Editor suggested to better describe the limitations of the study. In particular, we included sample selection biases, validity and reliability of the outcomes, and the observational nature of the study precluding clarification of causality. We also included several other limitations to the study that were noted by the Reviewers, including: single item measure of burnout, not utilizing validated self-report measures, no prior definition of “burned out” being provided to the survey participants.
5. The Editor recommended incorporating “Research in context” into Introduction and Discussion. We updated our manuscript to remove this section and follow PLOS ONE guidelines and the Editor’s recommendation.

**Reviewer #1**

**General:**

1. We expanded the Limitations section to include the valuable suggestions highlighted by the Reviewers and the Editor.
2. The Reviewer suggested to include demographic variables in the multivariate regression. We agree and have included all demographic information obtained from participants: provenience and occupation. These two variables were included in the multivariate regression, along with: burnout (binary), feeling pushed beyond training, adequate PPE, mental health support, receiving COVID-19-specific training, making life prioritizing decision, exposure to patient(s) diagnosed with COVID-19, and work impacting household activities because of COVID-19.

**Abstract:**

1. We thank the Reviewer for the appreciation of the geographical spread of our questionnaire. We believe that providing professional translations in several languages was greatly helpful, as well as including various HCP categories and populations that are not usually under the spotlight of the scientific community.
2. Following the Reviewer’s suggestion, we included the definition of burnout in the Methods section.

**Research in Context:**

1. “Research in Context” section was removed from the manuscript to follow the Editor’s suggestion and PLOS ONE guidelines.

**Introduction:**

1. We rectified the confusion between “COVID-19” and “SARS-CoV-2”.

**Methods:**

1. We thank the reviewer for the comment on geographical distribution and language translation.
2. The Reviewer argued that the study lacked validated self-report rating scales and instead used 40 questions based on expert opinion. We fully agree with the Reviewer and included this as an additional limitation of the study. We made the choice, in agreement with the experts that participated in the survey design and the pilot testing feedback, to omit the 22-item MBI. We shortened the mental health assessment to allow space for other questions related to the HCPs perception and workload during the pandemic.
3. Following the comment received from the Reviewer, we added a sentence to the Methods section to include the description of the collected demographic data: country of provenience and occupation.
4. The Reviewer argued that a single question on self-reported burnout seems like an unstable measure that would show high intra-individual variability. We agree and would have included the widely employed MBI burnout assessment if we anticipated such high participation from HCPs. During the pilot testing, we decided to reduce the number of potentially redundant questions, with the objective of increasing response rates (also impacting the type of demographic data being collected) in concert with mental health experts.
   The use of a single item questionnaire for self-reported burnout in the domain of emotional exhaustion is mentioned as one of the valid and reliable survey instruments to measure burnout in the link provided by the Reviewer.
5. The Reviewer questioned the fact that only the data gathered by the participants who responded completely was included in the statistical analyses. We agree with this suggestion to assess whether completers differ from non-completers in any way. A phrase has been added to the Statistical Analyses paragraph to state how the differences between completers and non-completers were insignificant. All variables included in the multivariate analysis were examined to assess possible differences. Here, the variables are reported with their Fisher’s exact test p values, with no difference between the completers and non-completers being statistically significant: burnout binary (*P*=0.4563), feeling pushed beyond training (*P*=0.554), adequate PPE (*P*=0.152), mental health support (*P*=0.8245), receiving COVID-19-specific training (*P*=0.1969), making life prioritizing decision (*P*=1), work impacting household activities because of COVID-19 (*P*=1).
6. The Reviewer noted that no definition of “burned out” was given to participants before the start of the survey. We understand that there may be significant differences in how burnout is perceived across different cultures and languages and included this as a study limitation.

**Results:**

1. We recognize the term “valid” can be misleading. Due to the nature of the online survey all answers were considered valid. We removed the term “valid” from the Results section.
2. We agree with the Reviewer’s comment on the need for a multivariate analysis to determine the factors independently associated with burnout. The forest plot in Figure 2 reveals the relationship between burnout and other factors using multivariate analysis.
3. We agree with the Reviewer’s concern on the lack of attempts to examine demographic associations with burnout. In order to address this limitation, we included the two demographic variables that were recorded, provenience and occupation, demonstrating significant differences for reported burnout across countries and occupations.

**Discussion:**

1. We included a citation to the statement kindly highlighted by the Reviewer.
2. We thank the Reviewer for pointing out the overarching message conveyed by our paragraph. We rephrased our findings in a more cautious manner, suggesting that burnout could be reduced, and further studies would be needed to confirm the initial findings.
3. The Reviewer noted that not all HCPs who worked during the SARS pandemic in Beijing demonstrated PTSS symptoms. We agree with this critical point and rectified that part of the manuscript accordingly.

**Reviewer #2**

1. We thank the Reviewer for their comment. We referenced the studies cited by the Reviewer in the Introduction section, which now better explains the necessity of our study.
2. The Reviewer requested a more detailed description of the methods used to identify potential participants and disseminate the survey. We expanded the Sample Population paragraph to include information about the recruitment strategy. Unfortunately, we did not record the number of social media groups that were contacted, and Facebook does not provide the number of individuals that saw each post. For these reasons, we cannot calculate or estimate the number of potential participants.
3. The Reviewer noted that occupations such as student, administrative staff, “not a HCP” were included in the questionnaire and Table 4. In the multivariate logistic regression, the occupations were grouped into 3 categories: medical doctors, nurses, and others. Students and administrative staff were categorized as “others”. “Not a HCP” was excluded from the study.
4. The Reviewer expressed concern about the impossibility of discussing burnout prevalence due to the missing representativeness of the survey respondents. We modified the Discussion and Conclusion sections accordingly, to refer only to the burnout level reported by the survey respondents.
5. We agree with the Reviewer’s concern about the reliability and validity of the translated scales, as well as the cut-off point of 5 that was considered for the conversion of reported burnout into a binary variable.
   Single item measure for the burnout dimension of emotional exhaustion has been proven to be the only aspect of burnout demonstrating global consistency [(pp. 118-121) doi 10.1007/978-3-319-52887-8_5]. For this reason, we felt confident in disseminating the survey in 18 languages with translations provided by certified professional translators. Other burnout domains would not have performed superior, as the Western concepts of depersonalization and personal accomplishment have been demonstrated to be inapplicable across different cultures. We also acknowledge the concerns coming out of the transformation of a 7-point Likert scale into a binary variable. West et al. have described the correlation between MBI scores and answers to the single item measure of the burnout domain of emotional exhaustion (doi:10.1007/s11606-009-1129-z]). The mean overall MBI score for emotional exhaustion by response to “I feel burned out from my work” (Table 2 in the cited article) describes a progressive increase in MBI score, consistent with the increase of frequency of the answers to the single item question. There is a clear and consistent cut-off between “Low and Average burnout” (response options 1 to 4) and “High burnout” (response options 5 to 7). The same cut-off was used to convert our 7-point Likert scale into a binary variable, as our objective was to differentiate between average and high reported burnout.
6. The Reviewer requested the number of respondents from HICs and LMICs and raised concerns about the appropriateness of this income subdivision. A total of 314 respondents were classified in LMICs and 1334 in HICs. Income based categorization is universally accepted, readily constructible and used in previous burnout assessments (for example doi:10.12688/gatesopenres.12779.3). We agree that we should state the importance of this classification more effectively in the study and the manuscript has been revised accordingly.
7. The Reviewer noted how burnout is assessed in 3 dimensions: emotional exhaustion, depersonalization, and personal accomplishment. We corrected our definition of burnout replacing “low personal achievement” with “personal accomplishment”.
8. The Reviewer noticed how the comparison between nurses and physicians reported in the text (OR=1.47) were different from those in Figure 2 (OR=1.12). Results of the multivariable regression analysis were correct in Figure 2 (RR=1.12, 95% CI=0.98-1.2). However, the manuscript erroneously reported the results of the bivariate analysis with emotional burnout as response variable (OR=1.47, 95% CI=1.12-1.92, P=0.006). The phrase has been removed from the manuscript due to lack of statistical significance.
9. The Reviewer noted how there were incongruences between the Funding Disclosure and the Acknowledgements section. We confirm that no specific funding was received for this work. The National Center for Advancing Translational Sciences, National Institutes of Health was mentioned because it supported Sandra Morales-Mirque who, in kind, offered her suggestions on the recruitment and dissemination strategies. Any reference to the National Center for Advancing Translational Sciences, National Institutes of Health has been removed.

We prepared a revised manuscript based upon these changes and submitted it’s clean and tracked versions through the online portal.

We are happy to respond to any further questions or suggestions from you or the Reviewers. I can most easily be reached by email at [lmorga5@uic.edu](mailto:lmorga5@uic.edu), office +1 (312) 413-9778, or by cell phone at +1 (617) 407-2410.

Thank you again for the opportunity to submit a revision.

Sincerely,

Luca A. Morgantini, M.D.
